# Soluble Recombinant Hemagglutinin Protein of H1N1pdm09 Influenza Virus Elicits Cross-Protection Against a Lethal H5N1 Challenge in Mice

**DOI:** 10.3389/fmicb.2019.02031

**Published:** 2019-09-04

**Authors:** Shinya Yamada, Atsuhiro Yasuhara, Yoshihiro Kawaoka

**Affiliations:** ^1^Division of Virology, Department of Microbiology and Immunology, Institute of Medical Science, University of Tokyo, Tokyo, Japan; ^2^Department of Pathobiological Sciences, School of Veterinary Sciences, Influenza Research Institute, University of Wisconsin-Madison, Madison, WI, United States; ^3^Department of Special Pathogens, International Research Center for Infectious Diseases, Institute of Medical Science, University of Tokyo, Tokyo, Japan

**Keywords:** influenza, vaccines, recombinant hemagglutinin protein, intramuscular immunization, cross-protection

## Abstract

Currently, influenza vaccines are produced using embryonated chicken eggs. Recently, recombinant influenza vaccines have been developed as a potential alternative to egg-grown vaccines. In this study, we evaluated the efficacy of soluble recombinant hemagglutinin (HA) protein produced in human cell culture (Expi293F cells) as an influenza vaccine against homosubtypic and heterosubtypic influenza virus challenges in mice. Mice were immunized intramuscularly with purified soluble HA protein of H1N1pdm09 virus and then challenged with a lethal dose of H1N1pdm09, seasonal H3N2, or highly pathogenic avian influenza (HPAI) H5N1 virus. Vaccinated mice showed better morbidity than mock-vaccinated mice following H1N1pdm09 challenge. By contrast, all mice died following H3N2 challenge. Interestingly, all vaccinated mice survived challenge with H5N1 virus, whereas all mock-vaccinated mice died. These results suggest that intramuscular immunization with recombinant HA proteins produced in Expi 293F cells could be of value in influenza vaccine strategies.

## Introduction

Vaccination is the main public health strategy used to prevent and control influenza. Influenza vaccines are currently produced by propagating selected vaccine seed viruses in embryonated chicken eggs. However, recent vaccine seed viruses have changed their antigenicity through egg adaptation, causing reduced vaccine effectiveness (Katz and Webster, 1989; Wang et al., 1989; Rajakumar et al., 1990; Skowronski et al., 2014). Cell-based vaccine production may offer a solution to this problem. Recently, recombinant proteins have been produced in mammalian cells, insect cells, plant cells, and *E. coli* as an alternative strategy for vaccine development (Lin et al., 2008; Wei et al., 2008; Biesova et al., 2009; Chiu et al., 2009; Shoji et al., 2009; Song et al., 2009; Cornelissen et al., 2010; Kalthoff et al., 2010; Du et al., 2011; Khurana et al., 2011; Chen et al., 2013; Prabakaran et al., 2013; Wohlbold et al., 2015; Ge et al., 2016; Sim et al., 2016; Dunkle et al., 2017). As an added advantage, the absence of egg proteins in cell-based vaccines eliminates the potential for egg-related allergic reactions. In addition, cell-based vaccines can be stably produced with a shorter lead time than the 6 months required for egg-based vaccine production, and production would not be affected by the supply of eggs (Milian and Kamen, 2015). A recent report showed that a recombinant influenza vaccine provided 30% greater efficacy than a standard inactivated influenza vaccine against influenza H3N2 in a clinical trial with a total of 9,003 recipients 50 years of age or older (Dunkle et al., 2017). However, in many of these studies, the recombinant proteins were produced by using a baculovirus expression system. A previous report showed that hemagglutinins (HAs) produced in 293T cells induce higher hemagglutination inhibition (HI) antibody titers compared with those produced in insect cells (de Vries et al., 2012). Expi293F cells, which were developed to produce a large amount of recombinant protein, are derived from the 293 cell line (Jain et al., 2017). We, therefore, used this cell line for our study to produce a recombinant HA protein. However, the vaccine efficacy of a recombinant HA produced in Expi293F cells has not been fully explored.

In many previous protection studies involving immunization with recombinant proteins, only the efficacy against the homologous challenge was examined. Although some studies showed cross-protection in mice against heterologous H5 virus challenge, the results were not obtained through intramuscular immunization of the recombinant HA protein but *via* intranasal infection with cold-adapted H1N1pdm09 influenza virus or baculoviruses that possessed influenza HA proteins (H1N1pdm09) (Jang et al., 2012; Sim et al., 2016). In addition, attenuated H5 viruses (not highly pathogenic H5 viruses) were used in those studies. One study has shown that intramuscular immunization with recombinant headless HA protein protects mice from heterologous virus challenge with H5N1 virus, but, again, attenuated H5N1 virus was used in the challenge experiment (Wohlbold et al., 2015). Therefore, the efficacy of the recombinant HA protein of seasonal influenza virus against heterologous highly pathogenic H5N1 virus challenges as a vaccine has not been fully studied. Accordingly, in this study, we sought to evaluate the efficacy of a soluble form of the recombinant HA protein of seasonal H1N1pdm09 virus, expressed in mammalian Expi293F cells, in terms of its cross-protection of intramuscularly immunized mice against heterologous virus challenges with highly pathogenic H5N1 virus and seasonal H3N2 virus.

## Materials and Methods

### Ethics and Biosafety Statements

Human blood was collected in accordance with protocols that were approved by the Research Ethics Review Committee of the Institute of Medical Science, the University of Tokyo. Written informed consent was obtained from all participants. All experiments with H5N1 viruses were performed in biosafety level 3 (BSL3) laboratories at the University of Tokyo, which are approved for such use by the Ministry of Agriculture, Forestry, and Fisheries, Japan. All experiments with mice were performed in accordance with the University of Tokyo’s Regulations for Animal Care and Use and were approved by the Animal Experiment Committee of the Institute of Medical Science, the University of Tokyo.

### Cells

Madin-Darby canine kidney (MDCK) cells were maintained in Eagle’s minimal essential medium (MEM) containing 5% newborn calf serum (NCS). Human embryonic kidney 293 cells were maintained in Dulbecco’s modified Eagle’s medium (DMEM) containing 10% FCS. Expi293F cells (Thermo Fisher Scientific), maintained in Expi293 expression medium (Thermo Fisher Scientific), were incubated on an orbital shaker platform rotating at 125 rpm at 37°C under 8% CO_2_.

### Viruses

Mouse-adapted A/California/04/2009 (MA-CA04; H1N1pdm09) (Sakabe et al., 2011), mouse-adapted A/Aichi/2/1968 (MA-Aichi; H3N2), and A/Vietnam/1203/2004 (VN1203; H5N1) were propagated in MDCK cells or eggs and titrated in MDCK cells.

### Cloning, Expression, and Purification of Recombinant Hemagglutinin Protein

A gene fragment encoding the HA protein of MA-Ca04 without the transmembrane or cytoplasmic tail domain was amplified by PCR and cloned into the pCAGGS vector with a six-His tag coding sequence at the C-terminus. Primer sequences are available upon request. The plasmid was transfected into Expi293F cells by using ExpiFectamine 293 (Thermo Fisher Scientific) according to the manufacturer’s protocol. At 4–6 days post-transfection, supernatants were cleared by low-speed centrifugation (900 rpm, 4°C, 5 min) and incubated with Ni-nitrilotriacetic acid (NTA) resin (Invitrogen) according to the manufacturer’s instructions. Subsequently, SDS-PAGE and western blot analysis with an anti-His-tag mouse monoclonal antibody (MBL, Medical & Biological Laboratories) were performed. The concentration of the purified soluble MA-Ca04 HA protein (sMA-Ca04HA) was measured by using a Pierce BCA Protein Assay Kit (Thermo Fisher Scientific) according to the manufacturer’s instructions.

### Immunofluorescence Assay

Twenty-four hours after transfection with plasmids, 293 cells were washed with PBS and fixed with 4% paraformaldehyde and permeabilized with 0.1% Triton X100. Cells were reacted with a mixture of anti-H1N1pdm09 HA mouse monoclonal antibodies (3E11C8, 5G10F, 7C2G7, 9C4C11), which were prepared in our laboratory, and with an anti-His-tag monoclonal antibody (MBL, Medical & Biological Laboratories). The cells were then incubated with Alexa Fluor 546 goat anti-mouse immunoglobulin G (Invitrogen).

### Enzyme-Linked Immunosorbent Assay

Ninety-six-well microtiter plates were coated with 2 μg/ml purified sMA-Ca04HA and then incubated with serially 5-fold diluted human or rabbit antibodies (starting at 5 μg/ml). We used two pdmH1-specific human monoclonal antibodies (10-5-64/6 and R4-5-72/1); an anti-H3HA human monoclonal antibody (1429D88/2), which was screened and established from human peripheral blood mononuclear cells (PBMCs) in our laboratory; CR9114, which broadly recognizes a conserved epitope in the stalk region; and an H5HA-specific rabbit monoclonal antibody (clone ID89, Sino Biological Inc.). After a 2-h incubation at 4°C, the plates were washed three times with ice-cold PBS, and then incubated with horseradish peroxidase (HRP)-conjugated rabbit anti-human IgG or mouse anti-rabbit IgG (Jackson Immuno Research) (1:2,000 dilution) at 4°C for 1 h. The plates were then incubated with OPD (*O*-phenylenediamine, Sigma) in PBS containing 0.01% H_2_O_2_ for 10 min at room temperature, and the reaction was stopped with 0.05 ml of 1 N HCl. Absorbance was determined at 490 nm.

### Enzyme-Linked Immunosorbent Assay for Mouse Sera

Serum samples were collected from vaccinated mice and mock-vaccinated mice 2 weeks after the second and third immunizations. Ninety-six-well microtiter plates were coated with 2 μg/ml purified sMA-Ca04HA or recombinant HA derived from either A/Perth/16/2009 (H3N2) or A/Vietnam/1203/2004 (H5N1) (Sino Biological). After being blocked with 5-fold-diluted Blocking One (Nakarai), the plates were incubated with serially 4-fold diluted serum samples. After a 2-h incubation at 4°C, the plates were washed three times with ice-cold PBS, and then incubated with HRP-conjugated rabbit anti-mouse IgG (1:2,000 dilution) at 4°C for 1 h. The plates were then incubated with OPD in PBS containing 0.01% H_2_O_2_ for 10 min at room temperature, and the reaction was stopped with 0.05 ml of 1 N HCl. Absorbance was determined at 490 nm.

### Immunization and Virus Challenge

Five 6-week-old female BALB/c mice (Japan SLC) per group were intramuscularly immunized with 10 μg of purified HA protein adjuvanted with 50 μl of AddaVax (InvivoGen) three times with a 10- to 14-day interval between vaccinations. As a control, mice in the non-immunized group were injected with the same volume of PBS. Serum samples were collected 2 weeks after the second and third immunizations. To assess the protective efficacy of vaccination with sMA-Ca04HA, mice were intranasally infected with 10 mouse median lethal doses (10 MLD_50_) of mouse-adapted A/California/04/2009 (MA-CA04; H1N1pdm09), mouse-adapted A/Aichi/2/1968 (MA-Aichi; H3N2), or A/Vietnam/1203/2004 (VN1203; H5N1) under anesthesia 2 weeks after the final boost immunization. Morbidity and mortality were monitored for 13 days after challenge. Mice with body weight loss of more than 25% of their baseline body weight were euthanized.

### Virus Neutralization Assay

Serum samples collected from three vaccinated mice or three mock-vaccinated mice, which were pretreated with a receptor-destroying enzyme (RED II; Denka Seiken, Tokyo, Japan), in duplicate, were serially two-fold diluted with MEM containing 0.3% bovine serum albumin (BSA-MEM) prior to being mixed with 100 TCID_50_ (50% tissue culture infectious doses) of mouse-adapted A/California/04/2009 (H1N1pdm09), mouse-adapted A/Aichi/2/1968 (H3N2), or A/Vietnam/1203/2004 (H5N1) at 37°C for 30 min. The mixtures were then inoculated into MDCK cells and incubated for 1 h at 37°C. BSA-MEM containing TPCK-treated trypsin was added to each well and the cells were incubated for 3 days at 37°C. The cytopathic effect (CPE) was examined, and the neutralization titer was determined as the reciprocal of the highest serum dilution.

## Results

### Generation and Characterization of Soluble Recombinant H1N1pdm09 Hemagglutinin Protein

**Figure 1:**
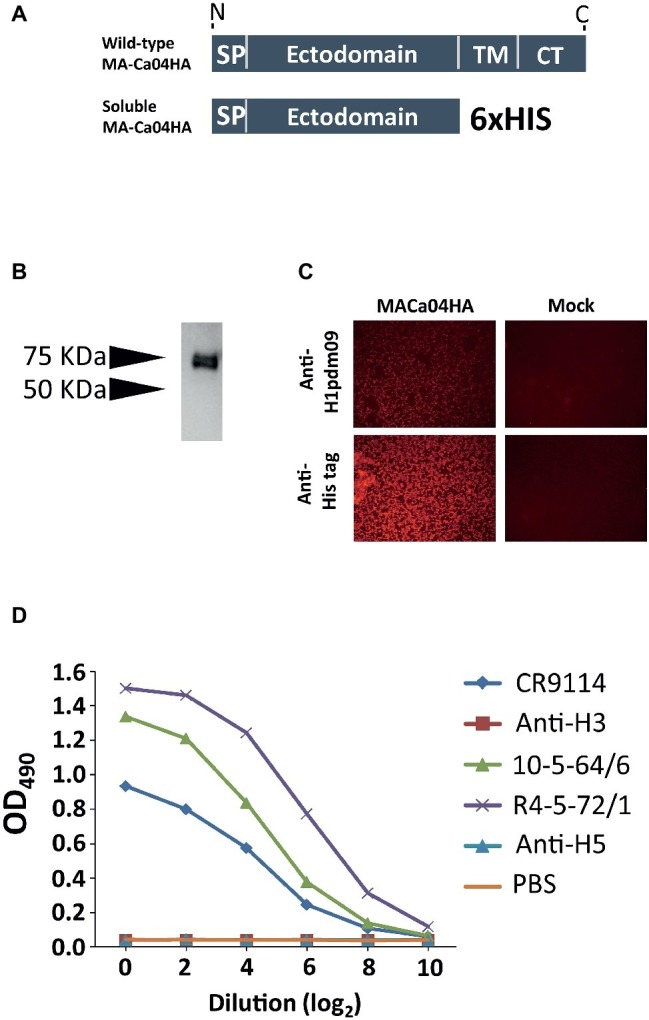
Characterization and purification of soluble recombinant HA protein. **(A)** Schematic of wild-type HA and the constructed soluble HA (SP, signal peptide domain; TM, transmembrane domain; CT, cytoplasmic domain). **(B)** The ectodomain of the HA from H1N1pdm09 virus with a His tag was expressed in Expi293F cells; purified soluble HA protein was obtained by means of SDS-PAGE under denaturing and reducing conditions. The expression of HA was analyzed by western blotting with an anti-His-tag mouse monoclonal antibody. **(C)** 24 h after plasmid transfection, 293 cells were fixed and stained with anti-H1N1pdm09HA monoclonal (3E11C8, 5G10F, 7C2G7, 9C4C11) and anti-His-tag monoclonal antibodies. **(D)** Binding properties of antibodies to soluble MA-CA04HA, as measured by ELISA. Two pdmH1-specific human monoclonal antibodies (10-5-64/6 and R4-5-72/1), an anti-H3HA human monoclonal antibody (1429D88/2), an HA-stalk-specific antibody (CR9114), and an H5HA-specific rabbit monoclonal antibody (clone ID89) were examined.

To express soluble recombinant H1N1pdm2009 HA (pdmH1HA) in mammalian cells, the ectodomain of mouse-adapted A/California/04/2009HA (MA-Ca04HA) (Sakabe et al., 2011) and a His-tag were cloned into the pCAGGS expression vector (Figure 1A), which was then transfected into Expi293F cells. The secreted HA was purified using Ni-NTA agarose chromatography. The expression and secretion of the soluble MA-Ca04HA protein with the His tag (sMA-Ca04HA) was examined by means of gel electrophoresis followed by western blotting using an antibody against the His-tag. The results showed that sMA-Ca04HA was successfully secreted into the supernatant with an expected molecular mass of 75 kDa (Figure 1B). Immunofluorescence analysis showed anti-pdmH1HA monoclonal antibody binding to the expressed HA protein, suggesting that the HA protein had folded properly (Figure 1C). We also examined the binding properties of several other antibodies to the soluble MA-CA04HA by using an ELISA (Figure 1D). We used two pdmH1HA-specific human monoclonal antibodies (10-5-64/6 and R4-5-72/1), which were screened and established from human PBMCs in our laboratory (Kubota-Koketsu et al., 2009); CR9114, which broadly recognizes a conserved epitope in the stalk region (Dreyfus et al., 2012); an anti-H3HA human monoclonal antibody (1429D88/2), which was also screened and established from human PBMCs in our laboratory (Kubota-Koketsu et al., 2009); and an H5HA-specific rabbit monoclonal antibody (clone ID89, Sino Biological Inc.). The two pdmH1HA-specific human monoclonal antibodies and CR9114 bound to sMA-Ca04HA, but the anti-H3HA and anti-H5HA monoclonal antibodies did not. These results suggest that sMA-Ca04HA retained the conformation and antigenicity of the HA protein of A/California/04/2009.

### Efficacy of sMA-Ca04HA as a Vaccine Against Lethal Infection in Mice

**Figure 2:**
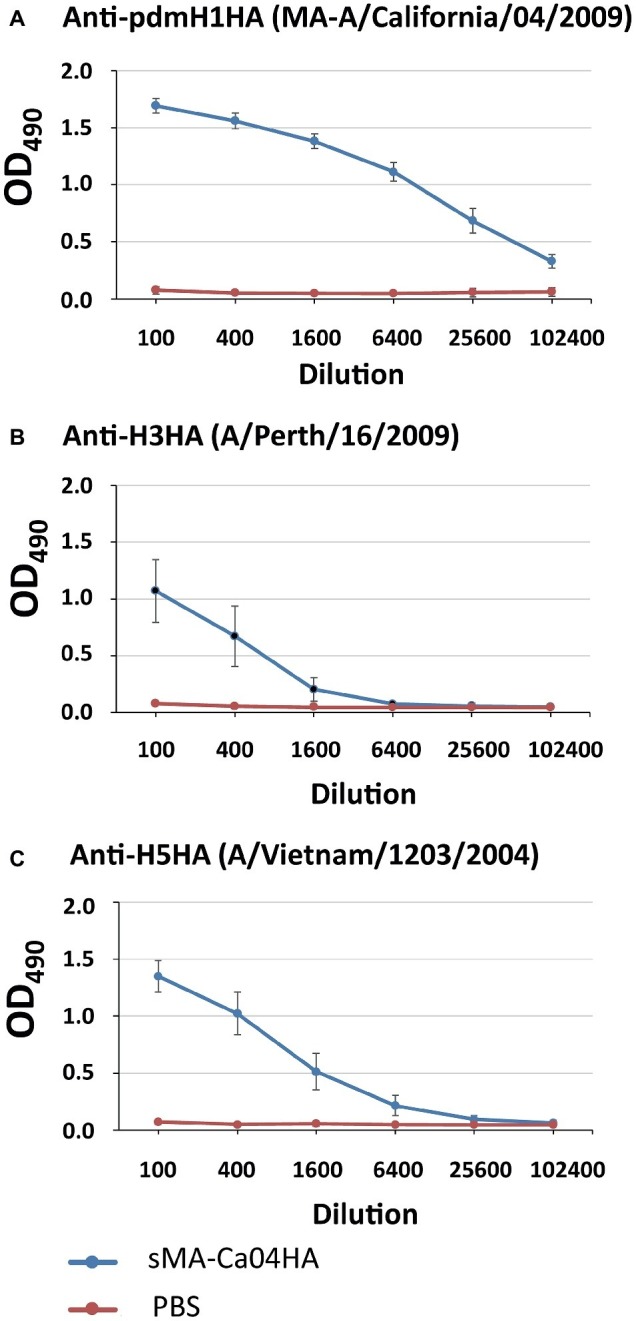
Antibody responses against influenza HA proteins in immunized mice. Sera from mice vaccinated with sMA-Ca04HA were analyzed by means of an ELISA against the purified HA proteins of H1N1pdm09 **(A)**, H3N2 **(B)**, and H5N1 **(C)** virus.

**Figure 3:**
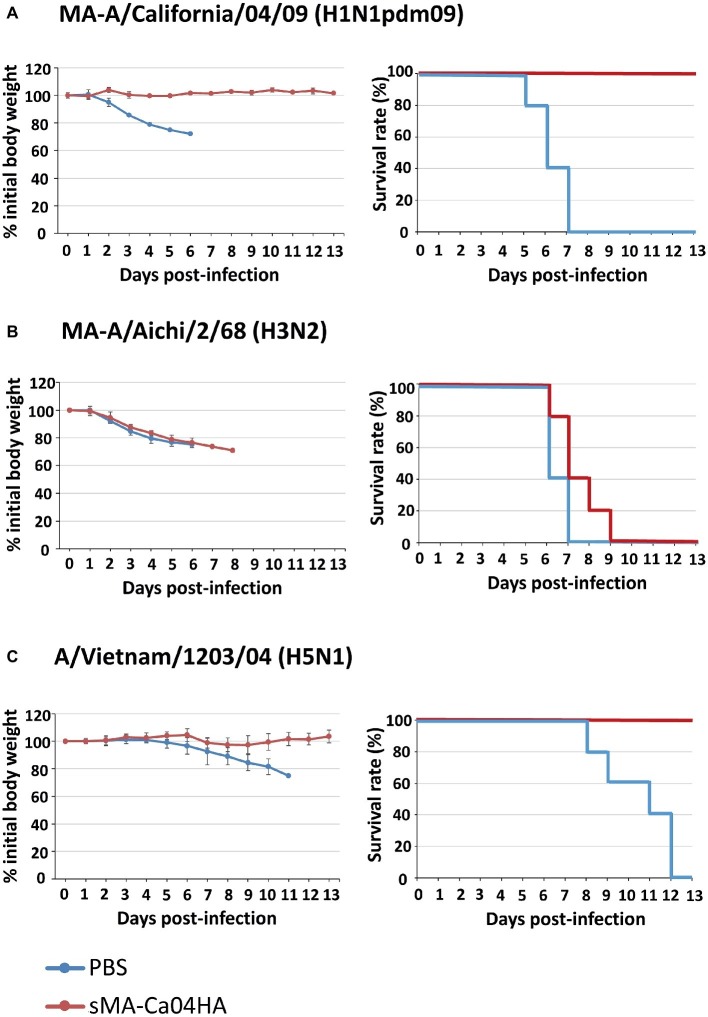
Body weight changes and survival of mice after virus challenge. Five mice per group were mock-immunized with PBS or immunized with sMA-Ca04HA three times with a 10- to 14-day interval between vaccinations. Two weeks after the final vaccination, the mice were intranasally challenged with 10 MLD_50_ of MA-A/California/04/09 **(A)**, MA-A/Aichi/2/68 **(B)** or A/Vietnam/1203/04 **(C)**. Body weight (left) and survival (right) were monitored for 13 days after challenge. Values are expressed as mean changes in body weight ± SD (*n* = 5).

To evaluate the immunogenicity of sMA-Ca04HA, naïve 6-week-old BALB/c mice were vaccinated intramuscularly with 10 μg of purified sMA-Ca04HA protein adjuvanted with 50 μl of AddaVax (InvivoGen) (Goff et al., 2013) three times with a 10- to 14-day interval between vaccinations. As a control, mice in the non-immunized group were injected with the same volume of PBS. Serum samples were collected 2 weeks after the second and third immunizations. We examined the seroreactivity induced by sMA-Ca04HA by using an ELISA against purified HA proteins (Figures 2A–C). Mice vaccinated with sMA-Ca04HA elicited high antibody titers against sMA-Ca04HA (Figure 2A). To determine whether immunization with sMA-Ca04HA could increase the breadth of reactivity, we analyzed heterologous seroreactivity against HA proteins from H5N1 and H3N2 viruses. The sera from sMA-Ca04HA-vaccinated mice showed reactivity in the ELISA to both H3HA and H5HA protein (Figures 2B,C). In contrast, mice injected with PBS elicited no antibodies reactive to the test HA proteins. To determine whether immunization with sMA-Ca04HA could induce neutralizing antibodies against homosubtypic H1N1pdm09 virus and heterosubtypic H3N2 and H5N1 viruses, we performed neutralization assays using sera obtained from three mice immunized with the recombinant H1N1pdm09 HA (Table 1). In the assay, we detected neutralizing activity against homosubtypic H1N1pdm09 virus but not against H3N2 or H5N1 viruses. To assess the protective efficacy of sMA-Ca04HA as a vaccine, we performed a viral challenge study (Figures 3A–C). Mice were infected with 10 MLD_50_ of mouse-adapted A/California/04/2009 (MA-CA04; H1N1pdm09), mouse-adapted A/Aichi/2/68 (MA-Aichi; H3N2), or A/Vietnam/1203/2004 (VN1203; H5N1) 2 weeks after their final boost vaccination. Upon challenge with MA-CA04, all mice vaccinated with sMA-Ca04HA showed no weight loss and survived, whereas mock-vaccinated mice lost more than 20% of their initial body weight and all of the mice died by day 7 post-infection (Figure 3A). In contrast, viral challenge with MA-Aichi/2/68 (H3N2) caused all vaccinated and mock-vaccinated mice to succumb to their infections within 9 days of challenge (Figure 3B). Interestingly, upon challenge with Vietnam/1203/04 (H5N1), all vaccinated mice survived and showed moderate weight loss, whereas the mock-vaccinated mice began to succumb to their infections at 8 days post-infection and all of these mice died within 12 days of challenge (Figure 3C). Our results indicate that this soluble recombinant HA protein mediates limited cross-protection against heterosubtypic influenza virus infection.

**Table 1:** Virus neutralization by serum of mice immunized with sMA-Ca04HA.

	Microneutralization titer using post-immunization mouse antisera against		
Mice inoculated with	MA-A/California/04/2009	MA-A/Aichi/2/1968	A/Vietnam/1203/2004
sMA-Ca04HA	2,560, 5,120, 10,240	<10, <10, <10	<10, <10, <10
PBS	<10, <10, <10	<10, <10, <10	<10, <10, <10

## Discussion

In this report, we showed that intramuscular inoculation of a recombinant Expi293F cell-based influenza vaccine candidate that comprised the ectodomain of H1N1pdm09 virus HA induced antibodies against the HAs of H1N1pdm09, H3N2, and highly pathogenic H5N1 viruses and provided protection against homosubtypic H1N1pdm09 and heterosubtypic H5N1 challenge in mice. Although sMA-Ca04HA immunization induced antibodies against H3HA protein, vaccinated mice were not protected from H3N2 challenge. H1 and H5 belong to group 1, whereas H3 belongs to group 2 of the phylogenetic groupings of hemagglutinin. Therefore, sMA-Ca04HA might induce antibodies against epitopes in the stalk region conserved among group 1 members that efficiently protect mice from challenge with viruses belonging to group 1. Interestingly, in neutralization assays using sera obtained from mice immunized with the recombinant H1N1pdm09 HA, we did not see any neutralizing activity against H5N1 virus in the sera, suggesting that the protection from the lethal H5N1 virus challenge afforded by the recombinant H1 HA is mediated by mechanisms other than virus neutralization.

Although our sMA-Ca04HA contained a His-tag, previous studies have shown that antibodies against His-tag are not generated in mice immunized with His-tagged recombinant proteins (Delaney et al., 2010; Shoji et al., 2011). We, therefore, assumed that antibodies against His-tag were not produced in our experiments and our results were not affected by the His-tag.

Recently, recombinant HA proteins have been produced on various platforms as an alternative strategy for influenza vaccine production. The glycosylation state of HA proteins differs depending on the cells in which they are expressed and this difference can affect the antigenicity of the proteins (de Vries et al., 2012). The report showed that HAs produced in 293T cells induce higher HI antibody titers compared with those produced in insect cells. In our study, we used Expi293F cells, which originated from the same 293 cell line as 293T cells. Therefore, we can assume that HAs expressed in Expi293F cells possess a similar glycosylation state and antigenicity to 293T cell-expressing HAs. Consequently, HAs produced in Expi293F cells may induce more effective antibodies compared with those produced in insect cells. The glycosylation state of the recombinant HA protein expressed in Expi293F might act advantageously to induce antibodies that target the conserved region of the HAs of H1N1pdm09 and H5N1 viruses and provide cross-protection mediated by mechanisms such as antibody-dependent cellular cytotoxicity. However, further studies are needed to better understand the influence of the glycosylation state of HA on its antigenicity and to understand which expression system is best suited for the development of a recombinant HA protein vaccine.

Previous reports have shown that intranasal infection of mice with cold-adapted H1N1pdm09 virus or baculovirus displaying the HA protein of H1N1pdm09 virus protected mice from H1N1pdm09 and H5 virus challenge (Jang et al., 2012; Sim et al., 2016). However, in these studies, attenuated viruses were used. By contrast, here we showed that intramuscular immunization with recombinant H1N1 pdm09 HA protein alone successfully protected mice from heterosubtypic highly pathogenic H5N1 challenge. Future mechanistic studies to understand how intramuscular immunization with recombinant HA could be sufficient to induce immunity to protect mice from heterosubtypic highly pathogenic H5N1 challenge will be important for vaccine development.

In conclusion, intramuscular immunization with Expi293F cell-based soluble recombinant HA protein induced protection against homosubtypic and heterosubtypic challenge in the same phylogenetic group. Our human Expi293F cell-based vaccine candidate thus offers a new strategy for influenza vaccine development. Further studies using other HA proteins from different subtypes would enhance the development of Expi293F cell-based vaccines. Further studies to understand the mechanistic basis of the immunity induced by the intramuscular administration of recombinant HA protein will aid in vaccine development.

## Author Contributions

SY designed the study and performed the experiments. SY, AY, and YK analyzed the data. SY and YK wrote the manuscript. All authors reviewed and approved the manuscript.

### Conflict of Interest Statement

YK has received speaker’s honoraria from Toyama Chemical and Astellas Inc.; has received grant support from Chugai Pharmaceuticals, Daiichi Sankyo Pharmaceutical, Toyama Chemical, Tauns Laboratories, Inc., Otsuka Pharmaceutical Co., Ltd., and Denka Seiken Co., Ltd.; and is a co-founder of FluGen.

The remaining authors declare that the research was conducted in the absence of any commercial or financial relationships that could be construed as a potential conflict of interest.
